# Development and Implementation of a Longitudinal Global Acute Care and Systems Strengthening Program

**DOI:** 10.5334/aogh.3385

**Published:** 2021-12-22

**Authors:** Cybil S. Stingl, Kyle J. Alexander, James M. Dittman, Noah J. Hillerbrand, Karishma Popli, Amira Dalmazio, Nancy Valencia-Rojas, Aline Baghdassarian, Sudha Jayaraman, Edgar B. Rodas

**Affiliations:** 1 Virginia Commonwealth University School of Medicine, Richmond, Virginia, US; 2 Department of Surgery, VCU School of Medicine, Richmond, Virginia, US; 3 Emergency Medicine and Pediatrics, Department of Emergency Medicine, VCU Health, Richmond, Virginia, US; 4 Program for Global Surgery, Department of Surgery, VCU Health, Richmond, Virginia, US

## Abstract

**Background:**

Increasing access to safe, timely, and affordable acute care in low- and middle-income countries is a worldwide priority. Longitudinal curricula on systems of acute care have not been previously described.

**Objectives:**

The authors aimed to develop a novel four-year longitudinal curriculum for medical students addressing systems development across multiple acute care specialties.

**Methods:**

The authors followed Kern’s six-step framework for curriculum design. After review of literature, a group of medical students and school of medicine faculty conducted a targeted needs assessment. Foundational goals and objectives were adapted from the 39 interprofessional global health competencies by the Consortium of Universities for Global Health. Educational strategies include didactic sessions, workshops, journal clubs, preceptorships, and community outreach. Clinical years include specialty-specific emphases, guided junior-level discussions, and a capstone project. Yearly SWOT and Kirkpatrick model analyses served as program evaluation.

**Findings:**

The Curriculum Council approved the program in July 2019. During the first cycle, the program matriculated 30 students from classes of 2023 (14) and 2022 (16). The first year produced 11 interactive sessions, 6 journal clubs, and 10 seminars led by 31 faculty and guest speakers; 29/30 students completed requirements; 87 evaluations reflected 4.57/5 content satisfaction and 4.73/5 instructor satisfaction. The 2023 cohort reported improved understanding of session objectives (3.13/5 vs. 3.82/5, p = 0.03). Free-text feedback led to implementation of pre-reading standardization and activity outlines.

**Conclusion:**

The Program was well-received and successfully implemented. It meets the needs of graduating medical students interested in leading global health work. This novel student-faculty collaborative model could be applied at other institutions seeking to provide students with a foundation in global acute care.

## Introduction

Global health has attracted substantial interest and participation amongst medical trainees. It is estimated that as of 2020, more than one-fifth of graduating medical students had participated in a global health experience [1]. These interests coincide with global recognition that access to safe, timely, and affordable emergency and surgical care in low- and middle-income countries (LMICs) should be a worldwide priority [2,3]. Academic institutions have started to respond to trainee interest and rising global emergency and surgical needs by increasing opportunities for early exposure to global healthcare, with multiple offering rotations in LMICs reflecting a short-term medical interventions model [4,5]. Initiatives at the fellowship level have been more comprehensive, recognizing that itinerant-driven international opportunities do not enable participants to play a future role in the leadership, organization, and development of sustainable long-term programs [6]. It is thought that global health initiatives require knowledge and implementation of health systems strengthening approaches to foster long-term positive impact on both trainees and the communities they serve [7]. Understanding and implementing such an approach requires a breadth and depth of global health training that core preclinical and clinical curricula do not provide [8].

A longitudinal curriculum for medical students that focuses on systems strengthening across multiple acute care disciplines has not been previously described or implemented. Longitudinal acute care curricula have been successfully developed in the past using the Kern Framework [9,10]. This framework, as first proposed by Kern et al. in 2010, is a six-step approach to curriculum development for medical education that progress through the following domains: (1) problem identification, (2) needs assessment, (3) goals and objectives, (4) educational strategies, (5) implementation, and (6) evaluation [11].

We aimed to apply the Kern Framework to develop a novel four-year longitudinal program for medical students at our institution that addresses systems development across multiple acute care specialties called the Acute Care Access and Systems Strengthening (ACCESS) in Low-Resource Settings Program.

## Methods

### (1) Problem Identification

Recognition of the need for a structured global acute care program for medical students at our institution required us to take a step back and evaluate the siloed efforts of student interest groups. Acute care faculty were routinely involved in mentorship at the request of student interest groups in student-led events relating to global surgery, emergency medicine, and anesthesia. We realized the extent of student interest when we collated the activities of global health interest groups. From 2018–2019, out of 90 registered medical student organizations at our institution, ten (11%) involved a global health focus, thirteen (14%) focused on acute care specialties, and three organizations pertained to both. We conducted a brief review of the published literature in April 2019 and found no papers on longitudinal medical school programs that covered global health, systems strengthening, and capacity building. This propelled us to create a structured curriculum in global acute care at our institution. Strong institutional programs such as I2CRP, HOMBRE, and fmSTAT already existed and served as models for a future program with varying global experiences and structure [12]. Student leaders of three acute care interest groups—the International Trauma Systems Development Program (ITSDP), the Panamerican Trauma Society (PTS), and the Global Surgery Student Alliance (GSSA)—met with acute care and emergency medicine faculty to discuss developing a four-year longitudinal program that would address student interest and help them adequately prepare for global health activities and leadership.

### (2) Needs Assessment

To carry out both a more formal needs assessment and determine the likelihood of student participation, student leaders of the three global acute care student interest groups arranged for their Class of 2022 members to sign up for the program’s preliminary student cohort. Nineteen students registered and committed to attend structured activities during the program’s development. During the 2018–2019 academic year, the student leaders organized a series of research, educational, and community outreach activities. The detailed record of student participation without formal structure comprised an observational needs assessment and served as evidence of the need for a curriculum (***Table 1***).

**Table 1:** Record of Preliminary Cohort Participation. Abbreviations: TEAM, Trauma Evaluation and Management. ^a^ Richmond Ambulance Authority Ride-Alongs are 12-hours each with a local emergency response and ambulance service.


EVENT DESCRIPTION	STUDENT INVOLVEMENT	DESCRIPTION

RESEARCH		

Participation in Global Health Research	16 students	Virginia Global Surgery Symposium: 5 oral presentations 2 poster presentations American College of Surgeons Clinical Congress: 1 oral presentation 3 poster presentations

**EDUCATION**		

Global Surgery Seminar Series	360 attendees over series duration	18 events with approximately 20 attendees on average

Virginia Global Surgery Symposium	27 Volunteers 250 symposium attendees	3-day event

Journal Clubs	41 attendances	3 events

TEAM Workshop	19 participants	1 workshop

Richmond Ambulance Authority Ride-Along^a^	6	6

**COMMUNITY OUTREACH**		

Hands-Only CPR Instructors	9 Student Instructors	2 Community Teaching Events

Stop the Bleed Instructors	17 Student Instructors	2 Community Teaching Events


### (3) Goal and objectives

Three faculty members with extensive global experience, an administrative program coordinator, and a team of six medical student leaders from the ITSDP, PTS, and GSSA student interest groups formed a curriculum development team. Initial efforts focused on balancing student desires with those identified from faculty real-world expertise. The goal of the curriculum was to provide medical students a longitudinal education focused on global healthcare systems and encompassing core competencies of acute care specialties such as surgery, emergency medicine, obstetrics and gynecology, anesthesiology, and critical care.

We adapted foundational objectives for the curriculum from 39 interprofessional global health competencies identified by the Consortium of Universities for Global Health (CUGH) [13]. We selected competencies at the Global Citizen Level as the essential learning objectives for the first and second years of the curriculum, corresponding with the preclinical years of medical training. The curriculum development team modified these objectives to better correspond to National Surgical Obstetric Anesthesia Plans and the application of a systems strengthening approach in the delivery of acute care [14]. We distributed the revised objectives over 11 didactic sessions so that students would achieve a common global healthcare systems background by the end of their preclinical years. These sessions and their objectives can be seen in ***Table 2***.

**Table 2:** Preclinical Curriculum Sessions: Focus Description and Objectives by Year.


SESSION TITLE	SESSION DESCRIPTION AND OBJECTIVE

M1 – FIRST PRECLINICAL YEAR (8 MONTHS)	

**Session M1-1: Global Burden of Disease (Domain 1)**	*Focus Description*: Through an interactive presentation and small group or case discussion, students will develop a basic understanding of the major causes of global morbidity and mortality and variations among high-, middle-, and low-income countries, especially in regards to acute surgical or medical disease. *Specific Objectives:* Identify the major causes of morbidity and mortality around the world as they pertain to acute care, and how risk factors and health determinants vary by world region.

**Session M1-2: Globalization of Health and Health Care (Domain 2)**	*Focus description:* Through an interactive presentation and small group or case discussion, students will develop a basic understanding of how globalization has impacted the spread of both chronic and acute disease, and be presented with an overview of healthcare systems that differ from our own. *Specific Objectives:* Describe how travel and trade contribute to the spread of communicable and non-communicable diseases, with special attention to diseases that require acute intervention.

**Session M1-3: Professional Practice: Access to Health care, Challenges in Local and Global Pre-hospital Healthcare Systems (Domains 2, 7)**	*Focus Description:* Through a presentation and open question session, representatives from Richmond Ambulance Authority will discuss the operation of a local prehospital care system and prepare students for a ride-along experience with their service. Next, faculty and student co-leader(s) will use an interactive presentation and small group or case discussion to discuss access to healthcare in emergencies and prehospital care in low-resource settings. *Specific Objectives:* Describe different national models or health systems for provision of health care. Develop a basic understanding of a local prehospital care system. Articulate barriers to health and health care in low-resource settings locally and globally, including in the delivery of emergency care in the prehospital care environment.

**Session M1-4: Global Research Ethics and Introduction to Journal Clubs (Domain 6)**	*Focus Description*: Through an interactive presentation and small group or case discussion, students will discuss common ethical issues that arise in global acute care and systems research, how it affects local communities, and what can be done to address these issues and build international collaborative partnerships. *Specific Objective:* Develop an understanding of common ethical issues and challenges that arise when working in service delivery and/or research of acute disease conducted in or applicable to low-resource settings, vulnerable populations, and within diverse economic, political, and cultural contexts.

**Session M1-5: Understanding Social and Environmental Determinants of Health (Domain 3)**	*Focus Description*: Through an interactive presentation and small group or case discussion, students will learn what social, economic, and environmental factors contribute to health in the acute setting. *Specific Objective* Identify major social and economic determinants of health, how they influence acute healthcare needs, and their effects on the access to and quality of health services.

**Session M1-6: Professional Practice and Preparing for a Global Health Experience (Domain 7)**	*Description*: Through an interactive presentation and small group or case discussion, students will discuss various clinical activities related to the delivery of acute care and surgery in low-resource settings. Students will also be presented with practical knowledge that directly prepares them for a global health experience. *Specific Objectives*: Become familiar with common health problems, especially those requiring acute care, and chief complaints in regions where VCU has participated with medical and surgical trips. Present a practical checklist to prepare students to embark on global health experiences.

**Session M1-7: Strategic Analysis for Healthcare in Low Resource Settings: The Basics of a Needs Assessment (Domain 11)**	*Focus Description*: Students will be presented with a systematic process for determining the acute care needs of a community, and a basic understanding of how a formal Needs Assessment fits into a planning and improvement process to strengthen healthcare systems. *Specific Objective*: Describe the steps for conducting a needs assessment at facility or health systems levels for care delivery in low resource settings.

**Session M1-8: Building Sustainable Emergency and Acute Care Programs in Low-Resource Settings (Domain 4)**	*Focus Description*: In this session, students will engage in a group discussion about two pillars of strengthening and building essential and sustainable emergency and acute care services: a skilled acute care and surgical workforce and the establishment of local partnerships. *Specific Objective*: Learn how the global acute care workforce shortage crisis impacts capacity building initiatives worldwide. Appreciate the importance of collaboration with a host or partner organization to assess an organization’s operational capacity.

**M2 – SECOND PRECLINICAL YEAR (5 MONTHS)**	

**Session M2-1: Understanding Health Policy in Low Resource Settings and Introduction to NSOAPs (Domain 9)**	*Focus Description*: Through an interactive presentation and small group or case discussion of the implementation of a National Surgical Obstetric and Anesthesia Plan (NSOAP) in Ethiopia, students will gain insight into the design, implementation, and evaluation of global surgery, anesthesia, obstetric services in low resource settings. *Specific Objectives*: Identify effective project management techniques used throughout NSOAP and other program planning, implementation, and evaluation. (Domain 9)

**Session M2-2: Social Justice: Strategies for Addressing Inequity in Low Resource Settings (Domain 8)**	*Focus Description*: Through an interactive presentation and small group or case discussion, students will learn about fundamental vulnerable populations; health and human rights; and health inequalities, especially as they relate to acute and critical care needs. *Specific Objectives*: Acquire a basic understanding of the relationships between health, human rights, global inequities, and acute care needs. Discuss strategies to engage marginalized and vulnerable populations in making decisions that affect their health and well-being in both the acute and chronic setting.

**Session M2-3: Sociocultural and Political Awareness (Domain 10)**	*Focus Description*: Through an interactive presentation and small group or case discussion, students will develop an understanding of how limited resources are assessed and utilized when planning national surgical plans. *Specific Objective:* Develop an understanding and awareness of the healthcare workforce crisis in the developing world, the factors that contribute to this, and strategies to address this problem. (Domain 8)


Learning objectives for the program’s third year explored material relevant to an acute care specialty of the student’s choice. Students could choose from five emphases to guide their third year: surgery, anesthesia, obstetrics, emergency medicine, or critical care. The third-year sessions addressed four to six core topics in a medical specialty context, including developing educational materials for LMICs, discussion of medical ethics in commonly encountered global acute care (e.g., scope of practice), and conducting specialty-specific field needs assessments. A portion of the third-year curriculum, including focus descriptions and specific objectives, can be found in ***Appendix 1***.

The final year of the curriculum emphasized students’ ability to utilize the knowledge gained to synthesize novel material and practice leadership and mentorship skills. The two primary goals for the fourth year included (1) completion of a capstone project containing a global or local acute care and systems-based component and (2) mentorship of junior students within the program.

### (4) Educational strategies

Multiple learning approaches including didactic sessions, small group workshops, journal clubs, preceptorships, and community outreach were incorporated into the preclinical years. The first year of the curriculum (2 academic semesters) contained eight interactive sessions and the second year (1 academic semester) contained three. The interactive sessions covered the modified CUGH objectives through didactic, small group, and discussion activities led by faculty and fourth-year students. Journal clubs held every month supplement the interactive sessions by engaging students with literature relevant to the same objectives, moderated by fourth-year students and a faculty member or resident physician. Students were also expected to attend monthly seminars by guest global speakers or substitute other approved activities (e.g., department global health grand rounds) to increase exposure and provide networking opportunities. An existing one-to-one clinical preceptorship (24 h. total) within the VCU School of Medicine was modified to pair students with global health-experienced clinicians for their introduction to clinical medicine during the second preclinical year. Students were also paired with international peers for two hours of discussion and asked to complete one ride-along within a local EMS system. Students were encouraged to become Stop the Bleed and Hands-Only CPR instructors to teach classes with community partners. A program coordinator monitored minimum attendance requirements for each category of activity.

During the third year (12 months) students participated in additional interactive sessions and activities specific to their emphasis of choice (Surgery, Anesthesia, OBGYN, or Emergency Medicine). Each emphasis contained at minimum a series of four to five interactive sessions hosted by faculty in each of the respective departments. Material is presented asynchronously and in multimedia format (online videos, readings, and discussion boards) to enable students to participate despite varying clinical schedules and locations. Additionally, students are expected to continue attending journal clubs throughout their third year when feasible.

We structured capstone projects as independent research by students, each paired with a faculty mentor. The students submit proposals during the third year and execute the project throughout their clinical education. Optional elective time is available for the students to exclusively dedicate time to their capstone project during the third (two weeks) and fourth years (four weeks). The results of these projects will be first presented at an annual program presentation day in August of the students’ fourth year, with the expectation that they will subsequently be submitted for international presentation or publication. Students from all years of the program will be able to attend, motivating junior students to envision their potential impact and proposals. Fourth-year students additionally serve as mentors, enabling them to revisit and master previously learned material acting as journal club moderators, teaching assistants during didactic sessions, and research mentors for junior students.

### (6) Evaluation

Assessment of student achievement and progress in the program is participation-based, and includes (1) detailed reflections written by students on their experiences at the end of the program, (2) completion of a capstone project by the fourth year and presentation at the Global Medicine and Surgery Student Research Day, with a minimum score of 70% of the points awarded by a faculty panel, (3) satisfactory evaluation from local partners during any field experience as communicated between host faculty and ACCESS program faculty, (4) participation in a minimum number of program sessions and journal clubs as previously described and documented below in ***Table 3***, and (5) completing a comprehensive assessment of global student competencies at the end of the fourth year. In part 3, students are evaluated using local student evaluation tools, if already in existence. Should these not be available, the program plans to soon implement a standardized global rotation student evaluation form.

**Table 3:** Passing Requirements of the ACCESS Program.


YEAR	REQUIREMENT

1	Attend 6 of 8 interactive sessions with completion of the corresponding pre- and post- session materials Participation in 2 of 6 journal clubs Attendance at 4 of 10 monthly global seminar series Participation in at least one community engagement event (e.g. instructing Hands-Only CPR or Stop the Bleed) with satisfactory evaluation from local partners

2	Attend 2 of 3 interactive sessions with completion of the corresponding pre- and post- session materials Participation in 2 of 6 journal clubs 24 hours of preceptorship with a global health experienced faculty preceptor Capstone project proposal

3	Participate in 4 specialty-specific interactive sessions with completion of any relevant pre- and post- session materials

4	Support faculty as a Senior co-leader for at least one session for junior students Present Capstone project at the Global Medicine and Surgery Research Day with a minimum score of 70% of the points awarded by a faculty panel Moderate at least one journal club for underclassmen Submit Capstone project for presentation or publication to at least one national or international platform Comprehensive assessments of the global competencies


The ACCESS program itself will be evaluated through a yearly SWOT (Strengths Weaknesses Opportunities Threats) assessment conducted jointly with program faculty and student leaders. Additionally, feedback surveys following the Kirkpatrick model of education evaluation will be completed before and after program sessions by the faculty leader and students. The Kirkpatrick framework assesses educational outcomes using four levels: (1) reaction (satisfaction with the program), (2) learning (changes in knowledge, attitude, and skills), (3) behavior (changes in behavior as a result of training), and (4) results (changes in the field or clinical setting) [15]. We have currently implemented incremental cycles of feedback for Kirkpatrick levels 1 and 2 using a modified logic model, whereby links between program resources, activities, outputs and outcomes are routinely examined [16]. The program will soon implement criteria and evaluation of Kirkpatrick levels 3 and 4, with the development of on-site learning objectives and evaluation methods led by international host programs in partnership with ACCESS faculty leaders.

## Results

### (5) Implementation

Multiple rounds of collaborative discussion among the development team and feedback from clerkship directors led to the final curriculum design. The VCU School of Medicine Curriculum Council reviewed and approved the curriculum formally in June 2019 as the Acute Care Access and System Strengthening (ACCESS) in Low-Resource Settings Program, with an official start date of Fall 2019. Sixteen of nineteen students from the Class of 2022 Preliminary Student Cohort who had participated in related activities elected to formally join the program by continuing participation in the structured curricula.

The first official application cycle of the ACCESS Program received interest from 67/184 (36%) of the Class of 2023 and 21 formal written applications in Fall 2019. The program accepted 14 students through a blinded review and scoring by the entire curriculum development team. Faculty scores were weighted more heavily than those of student leadership. Of the 14 students accepted to the program, 10 (71%) had previous exposure in a field of international medicine ranging from participation in short-term medical missions to grant-funded public health research.

A total of 23 faculty from the fields of surgery (n = 8), emergency medicine (n = 7), anesthesia (n = 3), internal medicine (n = 2), obstetrics-gynecology (n = 1), pulmonary and critical care medicine (n = 1), and hematology/oncology (n = 1) volunteered as instructors, journal club moderators, seminar leaders, and clinical preceptors. While only 13 of these faculty were needed as clinical preceptors for second-year students, two additional faculty volunteered but were not needed. One anesthesia resident moderated a journal club. An additional eight guest speakers delivered seminars and participated in journal clubs; these included two surgical specialists, an anesthesiologist, an obstetrician-gynecologist, a respiratory therapist, a surgical NGO CEO, and the founder of a global health nonprofit. The student leadership team, four core faculty members, and a departmental coordinator provided administrative support.

The first full formal academic year of the program (2019–2020) included 16 of the 19 students from the preliminary cohort who matriculated directly into the program’s second year and the 14 students accepted into the program’s first year. The students received a total of 11 interactive sessions, 6 journal clubs, 10 global seminars, and all second-year students received a clinical preceptor placement with program faculty. Annual program requirements were completed by 13/14 students in the Class of 2023 and 16/16 students in the Class of 2022. Additionally, students participated in three community engagement activities teaching Hands-Only CPR and Stop the Bleed to the local community.

### (6) Evaluation

For the duration of the Fall 2019 semester, which marked the first semester of the formal curriculum, Kirkpatrick levels 1 and 2 (satisfaction and knowledge) were surveyed using a 1–5 Likert scale in Class of 2022 and 2023 cohorts via voluntary and anonymous course evaluations emailed after all seminars, workshops, and journal club sessions. Paired T-test was applied to compare pre and post session objective-understanding scores. Unpaired T-test was used to compare remaining measures of content and instructor satisfaction. Voluntary student free-response feedback provided in these course evaluations was utilized to guide incremental improvements in subsequent sessions.

**Figure 1:**
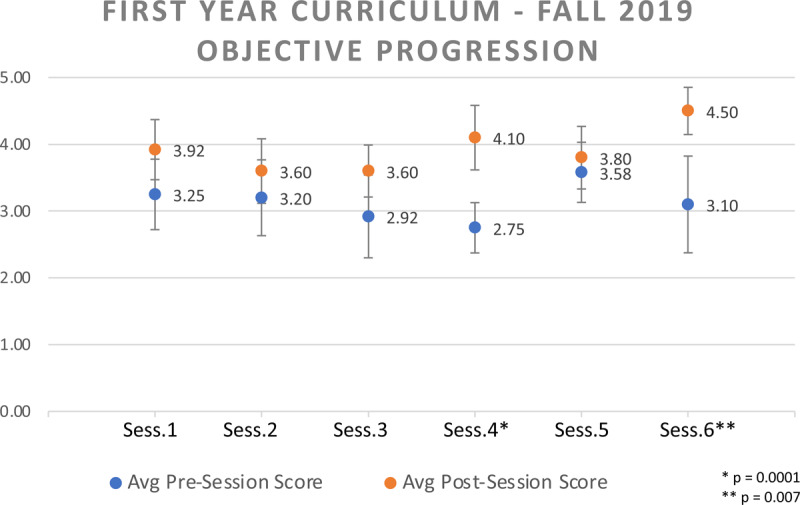
Fall 2019 Semester Sessions Objective Understanding Scores. In the first fall semester of the ACCESS curriculum, students improved in self-reported understanding of all session objectives (3.13/5 vs. 3.82/5, p = 0.03), with the overall significance driven by evaluations of objectives learned in Session M1-4: Global Research Ethics and Introduction to Journal Clubs and Session M1-6: Professional Practice and Preparing for a Global Health Experience.

Eighty-seven voluntary session evaluations were received over the course of the Fall 2019 semester, with 63 (72%) of these received from the Class of 2023. A paired T-test was applied to the 63 session evaluations and returned with an average content satisfaction of 4.57/5 and instructor satisfaction of 4.73/5. Overall the 2023 cohort gained significant improvement in self-reported understanding of all session objectives (3.13/5 vs. 3.82/5, p = 0.03), with the overall significant difference driven by evaluations of two of the six sessions (***Figure 1***). Free-text feedback was provided in 52 evaluations (60%). Based on free-text feedback, incremental session improvements in Fall 2019 included the standardization of pre-reading and activity outlines.

## Discussions

The ACCESS in Low Resource Settings Program was well-received and successfully implemented with strong student and faculty participation. It meets the needs of graduating medical students to have an understanding of healthcare systems before taking on leadership roles in global health through emphasis of CUGH competencies. Students within the program’s first semester improved in their self-reported confidence in program objectives, and their feedback led to improvements in curriculum delivery.

Existing global health curricula tend to be short-term in nature (like the four-week global health and surgery elective developed by Hugar et al.), focus on single-specialty care (such as the multi-year elective curriculum at Duke focussed on global surgery), or act at the postgraduate level when addressing the topics of capacity building and systems development (such as the global acute care surgery fellowship at Vanderbilt University) [17,18,19]. The ACCESS program is unique because it is longitudinal, teaches system development strategies, and is targeted towards the provision of multiple acute care specialties. Multiple disciplines in acute care, including emergency medicine, anesthesia, surgery, obstetrics, and critical care, were ideal for inclusion into the ACCESS program as each requires similar health system resources, face similar challenges, and are complementary in practice. This follows a broader trend within the global health community calling for integrated planning and collaboration between all specialties, ensuring proper resource allocation and chronic implications of acute care needs [20]. This is especially pertinent to understanding level 4 of the Kirkpatrick model as the implementation of strategies to strengthen global acute care systems will only be fully understood in the context of longitudinal primary care. It is also pertinent to understand the impacts of modifying secondary or tertiary acute care systems as inherently impacting local primary care via communication, transportation, referral, and counter-referral mechanisms. As these specialties are not practiced in siloes, further incorporation or partnership with primary care global health programs would be beneficial to the ACCESS Program.

The collaboration of several hospital departments allows for the flexibility students require as they determine the direction of their future careers while providing the comprehensive perspective required to become future leaders in capacity building. Curriculum development through utilization of the Kern Framework and student-faculty collaboration can be replicated at other institutions seeking to provide medical students with a similar comprehensive multispecialty background.

COVID-19 changes at the institutional level have impacted planned program events. While a majority of planned sessions can be held remotely, the current epidemic presents major barriers to student participation and leadership of community events as well as opportunities that require international travel. While limited by these constraints, evaluation of students by hosting partners within LMICs (part 3 of program evaluation) has been limited, which has also narrowed opportunities for assessment, revision, and maturation of the evaluation criteria and processes themselves. Upon resumption of international student travel, international partners will be engaged in the development of on-site learning objectives and evaluation methods used in assessing levels 3 and 4 of the Kirkpatrick framework.

It is important to recognize that identifying, developing, and maintaining international partners may be the greatest challenge for many institutions hoping to implement similar programs. At our institution, numerous faculty had pre-existing, long-term partnerships with global projects and were actively engaged in global work prior to the development of the ACCESS program based on local needs in those areas. Faculty-curated connections were especially useful in the development and implementation of capstone projects for students with no prior international background and pairing students with international colleagues. Students with global backgrounds and connections were able to pursue projects in additional geographic regions. Overall, capstone projects were based in part on local needs and in part on the interests and skills of each student, with foci ranging from global systems-strengthening to education and innovation in specific acute care specialties. It is important to note that not all capstone projects included international partnerships and data, but applied concepts learned through program curricula to resource-restrained settings in the United States such as rural Appalachia or the use of prehospital care by marginalized populations.

An additional current limitation of the program is a lack of program funding for student travel or research projects. In the future, students aspiring to complete capstone projects requiring capital or participate in international electives will require additional funding and administrative support not currently in place.

Creating structured international opportunities within the program for exposure to acute care specialties and health systems development is a key next milestone. In addition to requiring financial support for student research and travel, such opportunities will need close faculty collaboration and supervision to ensure students are participating in a level-appropriate and sustainable manner. Program commitment to specific initiatives will be necessary to ensure longitudinal progress and maintenance of international partnerships. It is remarkable that despite a lack of current institutional funding towards these efforts, students in our program have already participated in surgical and clinical work in numerous low-resource settings including Ecuador, Rwanda, Honduras, Chile, and rural Appalachia, Virginia. While this program received no dedicated faculty time from the hosting school of medicine during its development, there are plans to formalize and strengthen ongoing support.

## Conclusions

We describe a multiyear, multispecialty curriculum for medical students interested in capacity building, systems strengthening, and the delivery of acute care specialties in low-resource settings. Our student-faculty collaborative approach and novel curricula are unique in literature and may be reproducible at other institutions. Response from administration, faculty, and students has been positive and strong, and the program continues to mature with cyclical feedback. We believe the integration of modified CUGH competencies, comprehensive multispecialty education followed by specialty-specific emphases, local and international community engagement activities, senior to junior mentorship, and self-guided capstone projects provides students a unique foundation that will support future careers in global health systems development. Opportunities such as these should be more widely incorporated into medical school curricula.

## Data Accessibility Statement

ACCESS course evaluations were reviewed with permission from the program directors for quality improvement. The authors used no external data.

## Previous presentations

15th Annual Academic Surgical Congress, Orlando FL; Poster Session; February 2–4, 2020. Virginia Regional Health Sciences Education Symposium, Richmond VA; Poster Session; February 28, 2020. CUGH: Consortium of Universities for Global Health, Virtual Conference; Poster Session; March 12–14, 2021.

## Appendix

**Appendix 1:** Third Year Session Objectives in the Surgery Emphasis.

ACCESS SURGERY EMPHASIS	

SESSION TITLE	SESSION DESCRIPTION AND OBJECTIVE

**Session M3-General Session: Designing a Meaningful Global Surgery Capstone Project**	*Specific Objectives*: Designing of capstone proposal allows students to apply and demonstrate their knowledge acquired during the ACCESS elective course. This session will teach students how to identify feasible potential projects, organize ideas, define research methodology, and design an action plan. The project outcomes will be presented on the VCU Research day. Students will be encouraged to draft abstracts, submit the results to national conferences and convene their project’s findings/outcomes into an academic manuscript. *Specific Objective*: The proposal will include a research question or project objective and its importance, relevant background information and prior work, planned methodology, and a feasible timeline for completion of the project. The student will have received feedback and approval by their faculty mentor before proposal submission. Students will also complete selected CITI Online Research Ethics training modules by the date of proposal submission.

**Session M3-1: Strategic Analysis for Health Surgical Services in Low Resource Settings: The Basics of a Health Needs Assessment and Situational Analysis**	*Focus Description*: Strategic analysis refers to the process of analyzing existing information to formulate a strategy. A Health Needs Assessment and Situational Analysis are tools used to identify and analyze community health needs and the broad societal context. The process helps not only on the identification and prioritization of health needs but also in planning actions upon unmet needs. Surgeons and acute care providers working in international settings must possess a basic understanding of how a formal community needs assessment fits into the strategic planning of interventions for local surgical systems. *Specific Objective*: Each learner will possess a foundational understanding of what is involved in a needs assessment and SWOT (strengths, weakness, opportunities, threats) Analysis and how they can be used to draft an effective project proposal.

**Session M3-2: Ethical Challenges for delivery of surgical care in low-resources settings abroad**.	*Focus Description*: Given the many ethical issues and considerations arise when providing essential and/or elective surgery procedures in low-resources settings abroad, this session will offer students the opportunity to learned and to explore options on how best to address them. Being aware of common ethical challenges is the first step for self- awareness of own and others’ actions and when facing ethical decision-making situations. We will discuss ethical challenges such as: Belief of ‘good enough’ or ‘better than what they had’ White savior complex in missions Patient selection and follow-up for elective surgeries in LMICs Dilemma of disposable vs reusable surgical materials Use of limited resources to help just one patient (e.g. CT scan) *Specific Objective*: Each learner will possess a solid awareness and foundational understanding of common ethical challenges that impact the delivery of essential and elective surgeries in low-constraints settings.

**Session M3-3: Basic Surgical Skills Valuable in Resource-Constrained Settings**	*Focus Description*: The ability to perform and adapt surgical clinical skills in limited-resource settings is essential to provide quality care. Adaptability includes the use of essential clinical skills in settings with limited surgical providers and medical supplies. Instrument handling knot tying and suturing techniques IV access infusion Chest tube insertion Bleeding control techniques *Specific Objective*: Each learner will learn basic surgical skills needed when participating in global surgery activities

**Session M3-4: Common Emotional Challenges When Facing Prevalent Surgical Conditions in Low-Resource Settings**	*Focus Description*: Discussion of common surgical scenarios will prepare medical trainees for common challenges (both practical and emotional) faced when working in resource-limited settings. During this session, students will have the opportunity to analyze cases that would mimic challenging and realistic situations and would allow them to explore negative emotions related to the lack of adequate resources. Orthopedic Injury (Frustration) Abdominal Mass (Floundering) Rabies (Futility) Hypovolemic Shock (Frustration) Acute Appendicitis (Failure) *Specific Objective*: Each learner will participate in surgery-related simulation cases commonly encountered abroad to explore emotions of how they might feel when they don’t have the resources they are used to having at their disposal.

**Session M3-5: Educational Opportunities and Challenges in Global Surgery**	*Focus Description*: During this session, students will understand different strategies to reach out to different audiences or populations groups in LMICs with the goal of facilitating knowledge transfer and exchange. Principles of “knowledge sharing” and “knowledge exchange” can be used to educate patients and their families, colleagues, health policymakers, community members on topics related to the delivery of surgical care. Students would understand that educational strategies are bidirectional, and the realities of the local communities must be taken into consideration when designing and delivering them (e.g. low literacy). Sharing knowledge can be possible through different methods. Software platforms that offer telehealth and online chat services Oral presentations and talks Written materials: pamphlets, pictographs, *Specific Objective*: Each learner will understand the importance of educating patients, families, and communities with the use of simple educational strategies.
